# The morphology and evolutionary history of the glenohumeral joint of hominoids: A review

**DOI:** 10.1002/ece3.4392

**Published:** 2018-12-30

**Authors:** Julia Arias‐Martorell

**Affiliations:** ^1^ Animal Postcranial Evolution Lab Skeletal Biology Research Centre School of Anthropology and Conservation University of Kent Canterbury UK

**Keywords:** evolutionary morphology, glenohumeral morphology, hominins, hominoids, locomotion, Miocene apes

## Abstract

The glenohumeral joint, the most mobile joint in the body of hominoids, is involved in the locomotion of all extant primates apart from humans. Over the last few decades, our knowledge of how variation in its morphological characteristics relates to different locomotor behaviors within extant primates has greatly improved, including features of the proximal humerus and the glenoid cavity of the scapula, as well as the muscles that function to move the joint (the rotator cuff muscles). The glenohumeral joint is a region with a strong morphofunctional signal, and hence, its study can shed light on the locomotor behaviors of crucial ancestral nodes in the evolutionary history of hominoids (e.g., the last common ancestor between humans and chimpanzees). Hominoids, in particular, are distinct in showing round and relatively big proximal humeri with lowered tubercles and flattened and oval glenoid cavities, morphology suited to engage in a wide range of motions, which enables the use of locomotor behaviors such as suspension. The comparison with extant taxa has enabled more informed functional interpretations of morphology in extinct primates, including hominoids, from the Early Miocene through to the emergence of hominins. Here, I review our current understanding of glenohumeral joint functional morphology and its evolution throughout the Miocene and Pleistocene, as well as highlighting the areas where a deeper study of this joint is still needed.

## 1 INTRODUCTION

The evolution of primate locomotion and, especially, ape locomotion is fundamental to the understanding of human origins. Its importance resides on a central question of the discipline: How and when did we start walking on two feet? Bipedalism is a defining feature of being humans, and the study of the evolution of primate locomotor behaviors, with an emphasis on how morphological variation throughout the skeleton may relate to function, is key to answering such question. Theories on the origin of bipedalism, which try to elucidate the locomotor behavior exhibited by the last common ancestor (LCA) between humans and chimpanzees, range from characterizing the LCA as gorilla and chimpanzee‐like (thus knuckle‐walking; e.g., Richmond & Strait, 2000); as largely arboreal and similar to orangutans today (e.g., Thorpe, Holder, & Crompton, 2007); or engaging in a more heterogeneous and generalized pool of arboreal behaviors (climbing, clambering, bridging) as seen in the living arboreal primates (e.g., Arias‐Martorell, Potau, Bello‐Hellegouarch, & Pérez‐Pérez, 2015c). It is unfortunate that fossil remains throughout primate and, in particular, ape evolutionary history are scarce, and thus, we lack the evidence to fully support any one theory.

Among nonhuman hominoids, the forelimb is critical to a diversity of locomotor behaviors, ranging from terrestrial knuckle‐walking to suspension to ricochetal brachiation, but is largely removed from locomotion in humans. Within the forelimb, the glenohumeral joint—the articulation between the scapula's glenoid fossa and the proximal humerus—is the primary joint involved in arm movement and the most mobile joint in the body in hominoids (including *Gorilla*,* Pan*,* Pongo*,* Homo,* and the hylobatid family). As such, it has been the focus of morphological and biomechanical studies for decades and its major external morphological features have been functionally associated with the use of certain locomotor behaviors in primates (Arias‐Martorell, Alba, Potau, Bello‐Hellegouarch, & Pérez‐Pérez, 2015b; Arias‐Martorell, Tallman, Potau, Bello‐Hellegouarch, & Pérez‐Pérez, 2015a; Arias‐Martorell et al., 2015c; Kagaya, 2007; Larson, 1993, 1995, 2007a; Rose, 1989).

Within hominoids, the high degree of glenohumeral mobility has been widely linked to the evolution of an upright body posture, also known as orthogrady, which involved the displacement of the scapula onto the back of a mediolaterally wide and anteroposteriorly shallow thorax (e.g., Andrews & Groves, 1976; Gebo, 1996, 2010; Keith, 1903, 1923; Ward, 2015). The scapular displacement resulted in a more mobile and less stable glenohumeral joint, which may move in all directions and may combine all possible movements, from flexion and extension to abduction and adduction, and axial rotation (Larson, 1993). It is important that in hominoids (as well as in the groups of nonhominoids that use suspensory behaviors), the study of the glenohumeral joint is particularly useful to explore the presence of below‐branch positional behaviors and assess their dependence on suspensory locomotion (Arias‐Martorell et al., 2015a,b,c; Larson, 1993, 1995). However, despite decades of research, there still remains several unanswered questions, misconceptions, and debate about, for example, the functional morphology of the hominoid glenohumeral joint, particularly with regard to the role of soft tissue, specifically the cartilage surrounding the glenoid cavity (i.e., glenoid labrum; Arias‐Martorell et al., 2015c; Patton & Thiboudieau, 2010), the adaptive role played by the morphology of the joint with regard to the locomotor behaviors exhibited by the primates, the timing and acquisition path of such morphology, and intraspecific morphological differences between species of primates, in particular hominoids, with varied frequency on the use of the same locomotor behaviors.

The aim of this article is, then, to offer first a comprehensive review of the morphofunctional aspects of the glenohumeral joint and their relationship to the different locomotor behaviors exhibited by primates, with special emphasis on the ape clade, including Miocene hominoids and hominins, and then discuss future questions and aspects of the research that still need undertaking. In detail, I will first provide a general review of glenohumeral morphology and function, including soft tissues, giving an overall assessment of the morphological and functional diversity across primates and how they generally relate to the main locomotor modes found within the order. The review will then discuss in‐depth key morphofunctional characters of the proximal humerus and the glenoid cavity focusing on hominoids and using other primate groups to highlight differences in morphology related to opposing locomotor behaviors (e.g., suspension vs. quadrupedalism). In particular, as these traits are also commonly used to infer locomotor behaviors in the past, I will address this topic in the following sections dealing with the locomotion and evolutionary history of Miocene hominoids and hominins. The final section will be, as mentioned, a reflection on areas of future studies and possible directions on the analysis of the glenohumeral joint.

## 2 THE GLENOHUMERAL JOINT: OVERALL MORPHOLOGY AND FUNCTION

**Figure 1:**
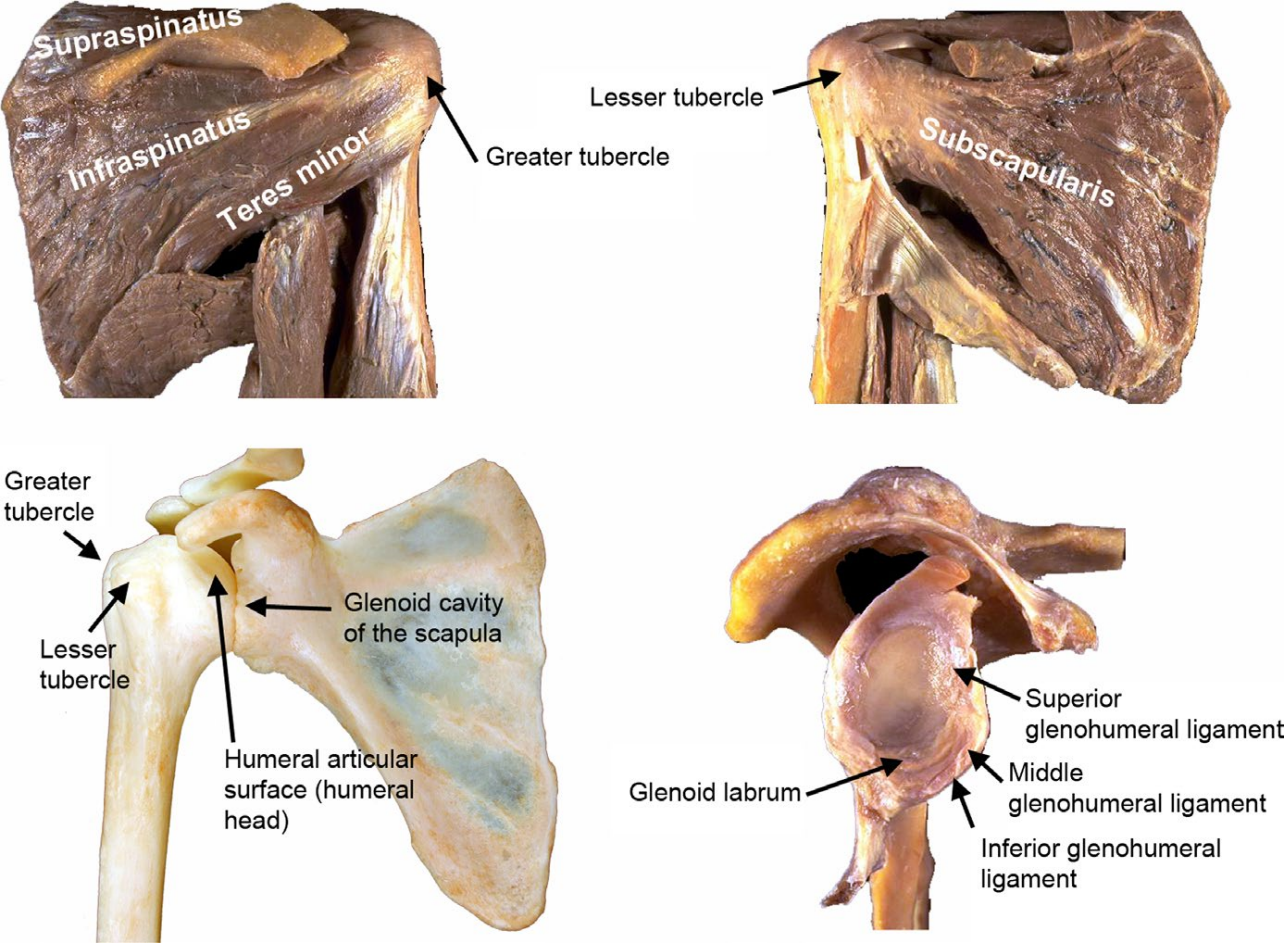
The rotator cuff muscles (supraspinatus, infraspinatus, teres minor, subscapularis) shown on a dissected human shoulder (top of the image). Osteology of the glenohumeral joint (bottom left). Soft tissue surrounding the glenoid cavity of the scapula, including the glenoid labrum and the remains of the capsule and the ligaments (superior, middle and inferior; bottom right). Pictures courtesy of JM Potau and A. Meri

The glenohumeral joint describes the articulation between the proximal humerus and glenoid cavity of the scapula (Figure 1). Important features include the rotator cuff muscles, which originate in the scapular blade (or fossae) and attach at the greater and lesser tubercles of the proximal humerus and provide stability and movement to the joint (Figure 1). There are four rotator cuff muscles: the subscapularis muscle, originating in the subscapular fossa of the scapula and inserting in the lesser tubercle; the supraspinatus muscle, originating in the supraspinous fossa of the scapula and attaching in the superior aspect of the greater tubercle; the infraspinatus muscle, originating in the infraspinous fossa and attaching in the lateral aspect of the greater tubercle; and the teres minor muscle, originating in the axial border of the infraspinous fossa and inserting distally to the infraspinatus insertion, also in the lateral aspect of the lesser tubercle (Figure 1; Testut & Latarjet, 1975; Rouviére & Delmas, 2002).

The glenohumeral joint has a flexible capsule that includes the glenohumeral ligaments (superior, medial, and inferior), which also aid to the stability of the joint. Finally, the glenoid labrum (the cartilage surrounding the glenoid cavity of the scapula) is the main cartilaginous structure with a potentially functional aspect, which is to extend the contact surface area of the glenoid, adding stability (Terry & Chopp, 2000; Figure 1). The morphology of the glenohumeral joint of primates is primarily the result of a compromise between mobility and stability. Primates engaging in quadrupedalism as their primary locomotor behavior favor more stable glenohumeral joints, whereas more acrobatic primates—particularly, hominoids—favor less stable glenohumeral joints, which result in increased mobility (Arias‐Martorell et al., 2015a; Larson, 1995; Rose, 1989), even though there have been some opposing views to that statement. Chan (2007) conducted a study on glenohumeral mobility and another on overall shoulder mobility in hominoids and monkeys (Chan, 2008) and concluded that in fact, hominoids had less mobile shoulders than monkeys. However, the study (2008) was conducted on sedated animals, which accounts for passive circumduction and not awake flexibility ranges, which might very well be extremely different and ultimately rendering hominoid shoulders more mobile. This study aligns with the latter standpoint (which is supported by the majority of research conducted on the subject; e.g., Arias‐Martorell et al., 2015a; Inman, Saunders, & Abbot, 1944; Larson, 1993; Veeger & van der Helm, 2007) and also follows the widespread convention that more mobility at the glenohumeral joint implies less stability, and on the contrary, less mobility is brought about by favoring more stability.

**Figure 2:**
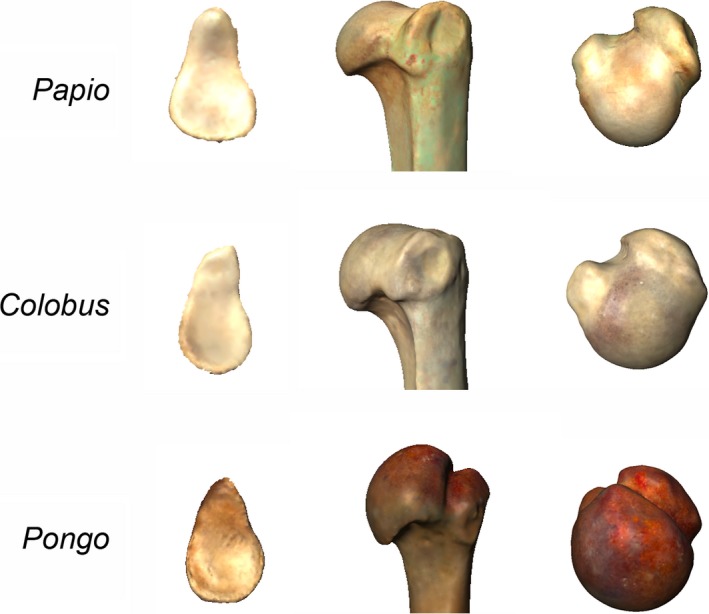
3D renderings of proximal humeri and glenoid cavities of a *Papio* (baboon), a *Colobus* (colobus monkey) and a *Pongo* (orangutan), showing the three main morphologies of the glenohumeral joint related to locomotion: terrestrial quadrupedal, with protruding humeral tubercles and a pear‐shaped glenoid cavity; arboreal quadrupedal, with a rounder humeral head and less protruding tubercles than the terrestrial quadrupeds; and suspensors, with a well‐rounded, globular humeral head and oval glenoid cavity

As such, terrestrial quadrupedal primates have the most stable glenohumeral joints, thus the least mobile. The proximal humerus and glenoid cavity are more proportionate in size, such that the glenoid cavity is larger and concave. This morphology allows for much of the proximal humerus to articulate with the glenoid during its full‐range motion. The proximal humerus is flattened in its cranial aspect, and its overall shape narrow and elongated relative to the more spherical shape of high‐mobility joints (seen in hominoids, e.g., see below). Furthermore, the tubercles are relatively large and protruding above the articular surface. Together, these features restrict mobility at the joint because of the lateral position of the scapula on the narrow thorax (Larson, 1988, 1993, 1995; Nakatsukasa, 1994; Preuschoft et al., 2010; Rose, 1989; Figure 2).

Arboreal quadrupedal monkeys display a similar morphology to terrestrial quadrupedal monkeys, although their morphology reflects stability as well as increased mobility. In particular, the globularity of the articular surface is greater, especially in its medial part, and the tubercles do not protrude as far from the humeral head (Larson, 1993, 1995; Rose, 1989). This configuration determines two functional regions within the articular surface, one in which the joint is fully flexed (protracted) and one in which the joint is fully extended (Larson, 1993). In extended positions, the region with which the glenoid cavity articulates with the humeral head is nearly spherical in outline, turning it into an almost a ball‐and‐socket joint (in that particular region), enabling relatively free mobility for feeding activities (reaching) and manipulative capabilities (as seen in some capuchin monkeys; Larson, 1993; Rose, 1989).

For the glenoid cavity, both terrestrial and arboreal quadrupedal primates exhibit a pear‐shaped morphology, produced by a ventral projection of the cranial margin of the facet into a more or less elongated lip structure, producing a high craniocaudal degree of curvature. For example, Old World Monkeys (cercopithecoids) tend to have slightly to strongly craniocaudally curved glenoid cavities, which favors flexion/extension movements at the glenohumeral joint over rotatory movements (MacLatchy, Gebo, Kityo, & Pilbeam, 2000; Figure 2). This morphology is typical of most quadrupedal animals and is probably a primitive condition (MacLatchy et al., 2000; Roberts, 1974).

Primates using below‐branch locomotor behaviors (suspension) have the least stable and most mobile glenohumeral joints. They typically show protruding and large, globular humeral articular surfaces, with relatively small tubercles lying well below the superior aspect of the humeral head, which increases the mobility and the motion range of the joint (Larson, 1993, 1995; Rose, 1989; Figure 2), to the extent that only 25%–30% of the humeral head is in contact with the glenoid cavity at any given time (Terry & Chopp, 2000) in this group of primates. A major functional feature of the glenohumeral joint of suspensory primates is the degree to which the tubercles of the proximal humerus are rotated to allocate for additional articular surface in the transverse plane (Corruccini & Ciochon, 1976; Fleagle & Simons, 1982; Larson, 1993; Rose, 1989), which results in an extensive, inflated articular surface that protrudes well above the superior aspect of the greater tubercle and it is directed medially (relative to the transverse axis of the elbow).

The glenoid cavity of suspensory primates exhibits an oval shape, which seems related to rapid limb motion with high acceleration increment when coupled with other elements such as elongated limbs, narrow scapulae and proximal concentration of musculature (Roberts, 1974). These primates also exhibit a moderate craniocaudal curvature, which allows a wide range of rotational shoulder movements (MacLatchy et al., 2000). It is in this group—which includes the suspensory hominoids—a small pool of Old and New World monkeys (e.g., *Presbytis*—Sumatran surili and *Ateles*—South American spider monkey) and also humans where the stabilization of the glenohumeral joint becomes a challenge. These primates achieve a substantial degree of abduction–adduction and axial rotation of the glenohumeral joint even when the joint is in a fully flexed position (Larson, 1993; Rose, 1989), which turns this into the most mobile joint of their body (Patton and Thiboudieau, 2010). Hence, these primates have to relay in additional sources for joint stabilization, both passive and active, which are reviewed in the following section.

### 2.1 Ligaments and musculature: function

The stabilization of the glenohumeral joint is achieved through a combination of passive—ligamentous and cartilaginous—and active structures—muscles. The role of the passive structures is not yet thoroughly understood, but the active structures, the rotator cuff muscles of the scapula that surround the joint with tendinous insertions, have been thoroughly studied (e.g., Ashton & Oxnard, 1963; Patton & Thiboudieau, 2010). Most data derive from electromyographical analyses, which have extensively recorded the activity and recruitment patterns of the rotator cuff muscles in primates (e.g., Inman et al., 1944; Jungers & Stern, 1981; Larson, 1993; Larson & Stern, 1986, 1987, 1989, 1992, 2013; Tuttle & Basmajian, 1978a,b).

During quadrupedal locomotion, either terrestrial or arboreal, Larson and Stern (1989) describe the supraspinatus muscle as being active during arm elevation, silent during swing phase, and active again during support phase. Other studies by the same authors demonstrate that the pattern is recurrent for baboons and macaques and suggest that this pattern could be common to all quadrupedal primates (Larson & Stern, 1989, 1992). The infraspinatus is, together with the supraspinatus, the main contributor to stabilization during the support phase of the quadrupedal gait. This muscle shows exclusive recruiting (along with the supraspinatus) during the swing phase of the gait (Larson & Stern, 2013). The infraspinatus is also involved in preventing humeral displacement during the support phase of quadrupedal walking (and knuckle‐walking, which I mention here as it is a modified form of quadrupedalism; Larson & Stern, 1987). More terrestrial primates seem to have more laterally facing insertions for the infraspinatus (Larson, 1995). In quadrupedal walking, there is a low level but high variability of recruitment of the teres minor muscle, which has prompted Larson and Stern (2013) to suggest that it is involved in finer rotatory adjustments of the glenohumeral joint rather than a key participant of the motion. At last, the subscapularis shows the same pattern of recruitment during quadrupedal walking than that of teres minor, thus seeming to also be involved in fine repositioning of the glenohumeral joint during gait (Larson & Stern, 2013). In African hominoids, it is especially important during the support phase of knuckle‐walking as well, when there is need to internally rotate the humerus to compensate for the shearing stresses caused at the glenohumeral joint due to the dorsal positioning of the scapula.

During suspensory locomotion, the supraspinatus acts as an abductor with the dual role of resisting humeral dislocation while assisting the deltoid in providing abductory power (Inman et al., 1944; Larson, 1993; Larson & Stern, 1986; Preuschoft, 1973; Preuschoft et al., 2010; Tuttle & Basmajian, 1978a,b). However, due to the protruding humeral head in primates using suspensory locomotion, the lever arm of the supraspinatus is reduced, posing a disadvantage that is solved by increasing the overall size of the supraspinatus itself (Fleagle & Simons, 1982; Larson & Stern, 1989, 1992; but see below for a more nuanced discussion on this topic). The infraspinatus acts as an abductor and lateral rotator, contributing to abduction through the middle phase of arm‐rising, as a primary synergist to the deltoid (Larson, 1993; Larson & Stern, 1986). The involvement in abduction seems to be related to the proximolateral oriented insertion of the muscle in the greater tubercle in hominoids. During locomotion, the infraspinatus is involved in bimanual and unimanual hanging and during the support phase of arm‐swinging, playing a specific role as a transarticular stress resistor by stabilizing the joint (Larson, 1993, 2013; Larson & Stern, 1986). During the support phase of arm‐swinging/suspensory behaviors, the teres minor is recruited as an adductor (tensile‐stress resistor) along with the infraspinatus—and there is barely any activity from the other rotator cuff muscles at this point (Larson & Stern, 2013). The teres minor also acts along with the teres major as a propulsor, and along with the caudal deltoid as an abductor during hoisting, also with a component of lateral rotation (Larson, 1993; Larson & Stern, 1986). The teres minor is differently recruited within suspensory hominoids, with great hominoids showing a small teres minor activity burst at the end of the swing phase, to tuck the elbow in, and lesser hominoids showing an early recruitment of the muscle during arm elevation, at hand release (Larson & Stern, 2013). Finally, the subscapularis primary role as medial rotator of the arm in primates—motion that can be combined with abduction or adduction—makes it extremely important during the support or “pull‐up” phase of climbing on a vertical trunk in hominoids (Larson & Stern, 1986, 1987). This medial rotatory function is also engaged in the support phase of true brachiation (Bello‐Hellegouarch et al., 2012; Larson, 1988). The subscapularis is divided in three portions, upper, middle and lower. During arm‐swinging, the lower and middle subscapularis are active during the first half of the medial rotational swing after hand release in arm‐swinging, while the upper part is silent. After that, re‐elevation of the humerus (abduction or elevation portion of the swing phase) is conducted by other rotator cuff components (Larson & Stern, 2013).

## 3 THE PROXIMAL HUMERUS: MORPHOLOGY AND FUNCTION IN DEPTH

Previous research has shown via 3D geometric morphometrics that there are two key aspects of the proximal humerus morphology that strongly reflect functional differences across different primate locomotor behaviors: the size and shape of articular surface and distribution of the insertions of the rotator cuff muscles on the humeral tubercles. Both of these aspects of morphology appear to more strongly reflect function rather than phylogeny (Arias‐Martorell et al., 2015a). This section will focus more deeply on these characters in hominoids, mentioning outgroups of New World and Old World monkeys either because of similarities with hominoids, or to highlight their differences.

### 3.1 Articular surface

**Figure 3:**
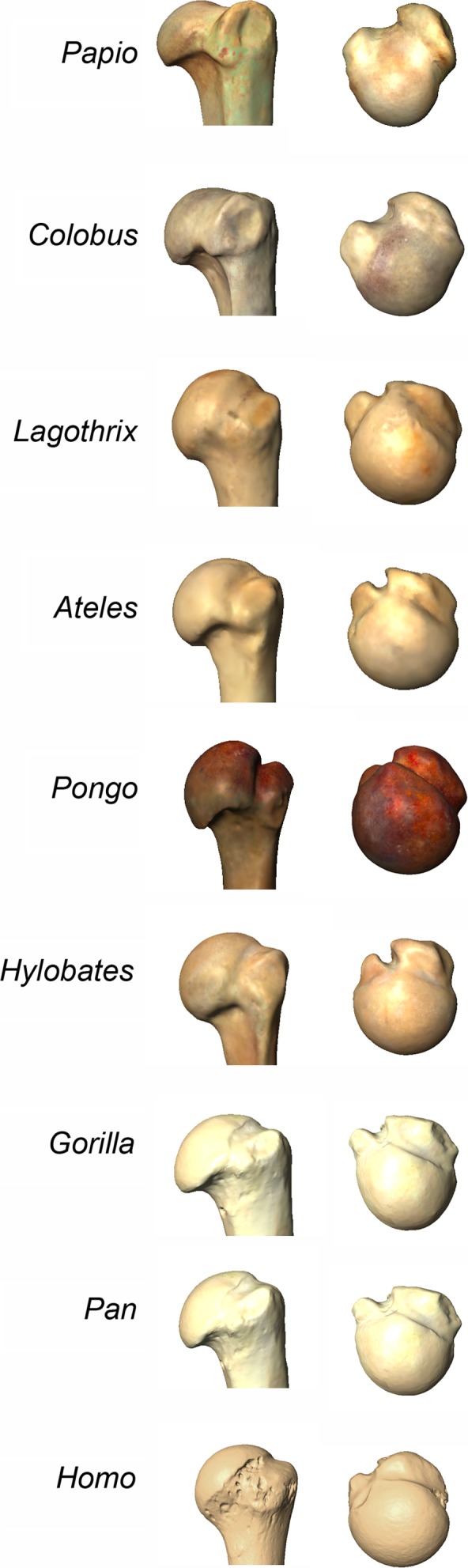
Comparative proximal humeral morphologies of all the groups mentioned, from the terrestrial quadrupedal *Papio* (baboon), the brachiator *Hylobates* (gibbon), to the terrestrial knuckle‐walkers *Pan* and *Gorilla* (chimpanzee and gorilla)

The major morphofunctional characteristics of the articular surface are the degree of globularity and the shape of the perimeter, which are largely indicative of the range of motion of the joint (Harrison, 1989; Larson, 1993; Rafferty & Ruff, 1994; Rose, 1989; Ruff & Runestad, 1992; Figure 3).

Primates that engage in quadrupedal locomotion (e.g., *Colobus—*colobus monkey, *Papio*—baboon) tend to show overall similar morphologies, with broad, oval‐shaped, and flat humeral heads that extended between the tubercles. However, differences can be observed regarding whether they are arboreal or terrestrial quadrupeds. Arboreal quadrupeds (e.g., *Colobus* and *Cebus*—capuchins) tend to exhibit slightly more rounded and inflated articular surfaces, whereas terrestrial quadrupeds (e.g., *Papio*) show flatter, most oval, and clearly extended between the tubercles articular surfaces. In general, quadrupedal monkeys (whether arboreal or terrestrial) offer a large articular surface of contact with the glenoid on the superior aspect of the humerus, which allows the joint to effectively transmit forces during the weight‐bearing phase of the gait (Larson, 1993; Preuschoft et al., 2010; Rafferty & Ruff, 1994; Rose, 1989; Figure 3).

*Lagothrix* (woolly monkeys), even though being primarily quadrupedal, exhibit an interesting intermediate morphology between fully suspensory primates and quadrupedal primates (Arias‐Martorell et al., 2015a). This group uses suspensory locomotion during only 11% of the time while traveling (Cant, Youlatos, & Rose, 2001, 2003; Kagaya, 2007), but their articular surface is still fairly globular and rounded, clearly differing from the protruding and extremely globular articular morphology of suspensory primates (see below), and, at the same time, clearly departed from the flattened and smaller articular surfaces of quadrupedal monkeys. The outline of the surface is oval‐shaped corresponding to its broader, more pronograde‐like locomotor repertoire (Cant et al., 2001, 2003; Kagaya, 2007; Figure 3).

In primates that engage in suspensory locomotion, there is a direct relationship between proximal humeral shape and amount of suspension they engage in (Arias‐Martorell et al., 2015a). Especially hylobatids (gibbons and siamangs), as they rely on brachiation as main locomotion mode (up to an 80% of the time; Fleagle, 1976; Hunt, 1991a; Michilsens, Vereecke, D'Août, & Aerts, 2009, 2010) and are the only group of primates that engage in its extreme form, ricochetal brachiation. Morphologically, then, hylobatids exhibit the most globular articular surfaces with circular perimeters, with proximal humeri well‐suited to perform a wide range of movements in the anteroposterior plane, mainly achieved by a lateral progression of the lesser tubercle (Figure 3). That allows the articular surface to expand in that direction, indicating a positive selection toward high‐mobility rates and wide‐range circumduction capabilities at the shoulder joint in this group (Arias‐Martorell et al., 2015a; Rafferty & Ruff, 1994; Ruff & Runestad, 1992).

*Pongo* (orangutans), who engage in varied forms of suspension (uni‐ or bimanual arm hanging, arm‐swinging and brachiation) show the most globularity on the proximal aspect of the articular surface (rather than its medial aspect), which is also expanded medially (toward the bicipital groove) instead of laterally as seen in hylobatids (Arias‐Martorell et al., 2015a; Figure 3). It is interesting that *Pongo* and the New World monkey *Ateles* (spider monkey) share the same proximal humeral morphology. This display of homoplasy between the two could be brought about by the varied use of locomotion of these two primates (from all forms of below‐branch locomotion to quadrupedalism, especially in *Ateles*), as well as a match in the amount of suspension they engage in without fully relying in this form of locomotion. One of the few proximal humeral shape‐focused studies available (Kagaya, 2007) found *Ateles* to be more similar to hylobatids, however, this study did not include *Pongo* in their comparative sample. Lack of proper comparative sample is an issue that will be discussed in subsequent sections.

*Gorilla* (gorillas) and *Pan* (chimpanzees) present a glenohumeral joint seemingly preserving all the traits necessary to engage in arboreal and suspensory locomotion (Arias‐Martorell et al., 2015a; Hunt, 1991b; Larson & Stern, 1987), despite their main locomotor behavior being a modified form of quadrupedalism known as knuckle‐walking (where they use of the back of the middle phalanges to make contact with the ground). Their humeral articular surface is still relatively big, globular and rounded, showing adaptations to a great range of motion. There appears to be a flattening of the central aspect of the joint (Figure 3), which instead of being a result of engaging in knuckle‐walking and of the compressive forces that could be acting at the joint during such activity, seems to be more advantageous for the functional demands of dealing with transarticular stresses during suspension for an effective diffusion of loads (Preuschoft, 1973; Preuschoft et al., 2010). This hypothesis, however, is at this time only valid for *Pan troglodytes* (common chimpanzee), species which we know only carry a 20% of their total body mass in their arms during knuckle‐walking (Kimura, 1985; Preuschoft, 1973, 2004). Data on forelimb weight‐bearing for *Gorilla* is needed to confirm or contest such hypothesis in this group.

Even though the proximal humeral morphology of *Pan* and *Gorilla* does not look exactly the same, more detailed studies focusing on their differences as well as finer studies on intraspecific variation of *Pan* and *Gorilla* species (for example, between *Pan troglodytes* and *Pan paniscus* (bonobos), and between *Gorilla gorilla* (lowland gorilla) and *Gorilla beringei* (mountain gorilla) and its relationship to differential locomotor behaviors, as studies conducted on other regions such as the hands or feet suggest exist; Dunn, Tocheri, Orr, & Jungers, 2014; Knigge, Tocheri, Orr, & McNulty, 2015; Tocheri et al., 2011) would have to be carried out to distill the differences between their glenohumeral morphology).

At last, modern humans, while still exhibiting rounded and protruding articular surfaces, show a morphological departure from the suspensory hominoids in that they display mediolaterally longer articular surfaces with an expansion in its medial aspect (Figure 3). The medial increase of articular surface could be related to functional demands of external rotation, which is important in retarding the contact between the greater tubercle and the acromion during the elevation of the arm in humans (Basmajian & De Luca, 1985; Inman et al., 1944). The mediolaterally longer heads seem to be related to the neutral (pendant) position of the lowered arm in humans, as the glenoid cavity mainly articulates with this region when the arm is downwards (Arias‐Martorell, Potau, Bello‐Hellegouarch, Pastor, & Pérez‐Pérez, 2012; Arias‐Martorell et al., 2015c). Small and precise movements to reposition the elbow and hands during manipulative activities could also occur in this area, favoring an enlarged surface for the glenoid to glide on.

### 3.2 Rotator cuff insertions

**Figure 4:**
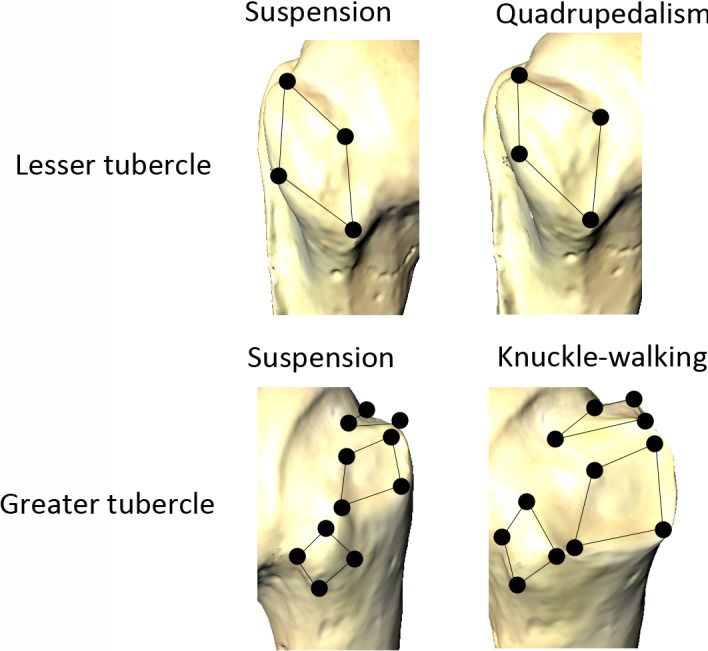
Distinct morphologies of the rotator cuff muscles attachments to the lesser and greater tubercles with respect to the locomotion used by the primates. Note the specific distribution of the greater tubercle insertions of the knuckle‐walking primates (*Gorilla* and *Pan*; Modified after Arias‐Martorell et al., 2015a)

The muscles of the glenohumeral joint insert in the rotator cuff in two locations: the greater tubercle (supraspinatus, infraspinatus and teres minor) and the lesser tubercle (subscapularis). The positioning of the insertion sites on the greater tubercle in different hominoids seemingly responds to the major stresses that apply to the joint, that is, compressive, shearing or tensile (Arias‐Martorell et al., 2015a; Figure 4).

As is the case with the articular surface, groups that rely or engage in relatively high amounts of suspensory locomotion exhibit certain similar characteristics. Hylobatids, *Pongo*, and *Ateles* exhibit the same pattern for the insertions on the greater tubercle: a line‐up in a proximodistal direction, mainly achieved by a lateral displacement of the teres minor insertion (Arias‐Martorell et al., 2015a; Figure 4). This pattern might be advantageous to secure the joint against tensile stresses: the teres minor muscle is activated in *Pongo* in overhead humeral adduction (Larson & Stern, 1986; Tuttle & Basmajian, 1978a,b), and the significant amount of lateral displacement of the teres minor insertion of highly suspension‐dependent taxa with respect to the articular may indicate a higher activity pattern for this muscle (Arias‐Martorell et al., 2015a).

Another feature of the rotator cuff insertions relating to the use of suspension in primates is the orientation of the infraspinatus insertion site. In hominoids in general, a higher degree of cranial/superior orientation of the muscle's facet seems to be related to the function of the infraspinatus muscle as the main stabilizer of the glenohumeral joint against forces pushing the humerus head away or along the glenoid cavity, mainly during pendant suspension and the support phase of arm‐swinging/brachiation (Larson, 1995; Larson & Stern, 1986; Roberts, 1974). Hylobatids display the least cranially orientated facet (Arias‐Martorell et al., 2015a; Larson, 1995), which is consistent with lesser apes being less dependent on the infraspinatus as abductor and lateral rotator during arm‐rising due to their low degree of humeral torsion (Larson, 1988, 1995) when compared to other hominoids and *Ateles*, which shows an orientation of the insertion similar to *Pongo*.

It is interesting to note that humans, who do not use any significant amount of suspension in their daily lives in general, also exhibit the above‐described pattern of the greater tubercle insertion. However, the glenohumeral joint of humans is indeed subjected to tensile stresses on the pendant limb. Differing from the suspensory primates, the greater tubercle of humans is overall smaller, with reduced insertion sites for the rotator cuff muscles, which possibly indicates a decrease of reliance on the active stabilizers of the glenohumeral joint due to a reduction in size of the rotator cuff, as there is no need to power a locomotor mode that would have this joint carry the total body weight of an adult (Arias‐Martorell et al., 2012). At the same time, this would have allowed for fast and precise manipulation movements. In particular, the supraspinatus muscle is fairly reduced in size in humans (Bello‐Hellegouarch, Potau, Arias‐Martorell, Pastor, & Pérez‐Pérez, 2013; Larson, 1995, 2007a; Potau et al., 2009; Roberts, 1974), which brings about a separation between the rotator cuff insertion areas and the humeral head. This happens for both the supraspinatus and the teres minor insertions and it might contribute to the increase of the leverage of both muscles to compensate for their relatively smaller size (Basmajian & De Luca, 1985; Inman et al., 1944; Potau, Bardina, & Ciurana, 2007; Potau et al., 2009; Roberts, 1974).

The main difference in the morphology of the greater tubercle insertions is seen in the knuckle‐walking *Gorilla* and *Pan*: contrary to all other hominoids, they exhibit a triangular disposition of the insertions, achieved by a lateral displacement of the infraspinatus insertion, and a closeness of the proximal and distal ends of the teres minor and supraspinatus, respectively (Figure 4). This may be advantageous to secure such mobile joint against the shearing stresses occurring during knuckle‐walking. *Gorilla* and *Pan* also show the greatest degree of cranial orientation of the infraspinatus facet, corresponding to their greater degree of humeral torsion (Larson, 1995) and to their higher dependence on the infraspinatus muscle to act as synergist to the deltoid in arm‐rising behaviors. The infraspinatus of these two taxa also shows an increase in size in respect to the other insertions, being the main cause of the distinctive triangular disposition of the rotator cuff insertions of the greater tubercle in the knuckle‐walkers. The infraspinatus has an important role in aiding the supraspinatus to assist the deltoid in arm‐rising behaviors, as well as exerting antigravitational forces during the stance phase of knuckle‐walking (Larson, 1993, 1995; Larson & Stern, 1986, 1987). Hence, these two muscles bear the responsibility of maintaining stability in a highly mobile joint (Larson & Stern, 1987).

*Pan* seem to generally exhibit enlarged rotator cuff muscle masses respect to the other hominoids (Kikuchi, Takemoto, & Kuraoka, 2012; Mathewson, Kwan, Eng, Lieber, & Ward, 2014; Oishi, Ogihara, Endo, Ichihara, & Asari, 2009; Potau et al., 2009), which could have resulted in a need of increase of insertion site space, contributing to their distinctive triangular disposition in the greater tubercle; however, this hypothesis has not been tested as such to date. Debate sparks whenever rotator cuff muscle mass enters the fray, especially regarding the question of whether hominoids have enlarged supraspinatus or infraspinatus muscles, with much of the data being derived from reported increased sizes of their attachment sites (ratios) at the scapula respect to each other (Bello‐Hellegouarch et al., 2013; Green, 2013; Roberts, 1974; Taylor, 1997; Young, 2008;). However, a recent study by Larson (2015a) suggests that muscle masses do not necessarily correspond to increased scapular fossae area, with variation between dorsal rotator cuff muscles mass not influencing the functional roles of such muscles, all of it showing a degree of dissociation between soft tissue properties and hard tissue morphology. In short, the functional roles in locomotion played by the individual rotator cuff muscles do not substantially differ among hominoids even if their masses do. The differences, then, would be brought about by the variation of each species in their specific locomotor repertoire (Larson, 2015a).

At the lesser tubercle, hominoids (including *Pan* and *Gorilla*) and *Ateles* exhibit the same narrow and spindle‐shaped morphology for the subscapularis insertion, in contrast to the rounded insertion exhibited by largely pronograde taxa (e.g., *Colobus*,* Cebus*,* Papio* and *Lagothrix*; Figure 4). As discussed above, the subscapularis muscle does not act as a unit in hominoids, but as three separated portions (lower, middle and upper; Larson & Stern, 1986; Larson, 1988, 1995). The shape of the insertion of the subscapularis in hominoids and *Ateles* reflects this differentiation, with the muscular fibers that originate from the most proximal part of the tendon (and therefore from the proximal part of the lesser tubercle) being involved in abduction and medial rotation, whereas those fibers originating from the distal parts being involved in adduction and medial rotation and not contributing to arm‐rising (Larson & Stern, 1986). The most caudal/distal fibers contribute to pulling the humeral head downward, toward the axilla (as a synergist to the infraspinatus). Such differentiation shows the versatility of this muscle in hominoids and *Ateles*, where it is extremely important in the stabilization of the glenohumeral joint during the support phase of climbing (Larson & Stern, 1986), as well as during the quadrupedal and knuckle‐walking stance phases (Tuttle & Basmajian, 1978b).

## 4 THE GLENOID CAVITY: MORPHOLOGY AND FUNCTION IN DEPTH

A recent study of the shape and function of the glenoid cavity determined that no morphological features are correlated to sex, activity level or side (Macias & Churchill, 2015). Among great hominoids, certain features of the glenoid cavity of *Pan* (laterally projecting articular glenoid rim and a central orientation of the deepest aspect of the fossa) could be related to vertical climbing (Macias & Churchill, 2015). These features might contribute to the stabilization of the glenohumeral joint at hind limb push‐off phase in climbing, where *Pan* protract their shoulders.

However, caution is needed when drawing conclusions from scapular glenoid shape until more studies are conducted, as the shape of the glenoid cavity seems to not be driven by locomotor constraints as much as the proximal humerus, according to a recent study by Arias‐Martorell et al. (2015b). For instance, in all the analyses orangutans exhibited morphological similarities of the glenoid cavity with *Lagothrix*, when these groups do not share the same locomotor repertoire. The shape of the glenoid cavity of orangutans was narrower and more curved than those of the other hominoids and exhibited a lip‐like reminiscent elongation of the cranial aspect, lightly resembling those of quadrupedal monkeys. In fact, the 3D GM analysis of the shape of the glenoid cavity, including all the hominoids and a suite of Old and New World monkeys, shows a complete overlap between *Pongo* and *Lagothrix* (Supporting Information Figure S1). However, the distinctive morphology of the glenoid cavity of *Pongo* could be related to a greater passive stabilization of the joint in abducted postures of the arm, permitting ball‐and‐socket joint contact in the medial and superior aspect of the proximal humerus (Kapandji, 2007). Such equivocal overlap between *Lagothrix* and *Pongo*, with the consequent relatively monkey‐like morphological affinities of the latter suggests that caution should be exercised when locomotor inferences are attempted based on the glenoid cavity alone, especially in cases where extinct taxa are involved (see below).

## 5 EVOLUTION OF HOMINOID GLENOHUMERAL MORPHOLOGY: THE MIOCENE APES

The amount of proximal humeri remains and scapular fragments with intact glenoid cavities from Miocene apes is scarce. This review considers the shoulder girdle and locomotor behavior of Miocene apes in general and, when possible, specifically their glenohumeral joint (either component).

The better‐known taxa from this period (both in general terms and in shoulder girdle remains numbers) are the Early Miocene (ca. 23–16 million years ago, Mya) apes, in particular *Proconsul*—including the recently erected *Ekembo* genus (*E. nyanzae* and *E. heseloni*), formerly considered proconsulids (McNulty, Begun, Kelley, Manthi, & Mbua, 2015)—and similar forms, such as *Nyanzapithecus* and *Afropithecus*. Early Miocene apes were powerful‐grasping, above‐branch quadrupeds/cautious climbers that retained a pronograde body plan and were morphologically generalized, showing no living ape‐like adaptations for below‐branch suspensory behaviors (Begun, Teaford, & Walker, 1994; Corruccini, Ciochon, & McHenry, 1975; Dunsworth, 2006; Fleagle, 1983; Morbeck, 1975; Rose, 1983, 1993; Walker & Pickford, 1983; Ward, 1997, 2015). When compared to extant taxa, Early Miocene apes show a combination of traits of arboreal quadrupedal Old World Monkeys and large New World Monkeys (spider, howler, and woolly monkey), and apes (Arias‐Martorell et al., 2015b; Rein, Harrison, & Zollikofer, 2011; Rose, 1983). They differ from earlier forms, such as the propliopithecoid *Aegyptopithecus*, in having the shoulder adapted to a wider range of loading, with a somewhat laterally projected acromion, an oblique spine and a cranially directed glenoid fossa, implying that (nonsuspensory) overhead positions of the forelimb could be easily achieved (Rose, 1983; Ward, 2015). The proximal half of their humerus is characterized by a shallow bicipital groove and a flat deltoid plane with well‐developed deltopectoral and deltotriceps crests, as observed in extant arboreal Old World monkeys (Napier & Davis, 1959; Rose, 1983). It is in the shape of the distal humerus that early Miocene apes most resemble hominoids, as well as in some aspects of the hands (Rose, 1983, 1988, 1992; Begun et al., 1994), which makes the combined features of the forelimb of these early apes suitable for an extended range of movement compared to earlier taxa (Rose, 1983).

For this period, there exists at least one evidence of a possible orthograde taxon, the early Miocene ape *Morotopithecus bishopi* (ca. 20 Mya, Uganda), which, according to the literature, exhibits orthograde features combining them with below‐branch locomotion (MacLatchy, 2004; MacLatchy et al., 2000; Nakatsukasa, 2008; Sanders & Bodenbender, 1994). While it is true that *M. bishopi* bears a great resemblance to modern hominoids in several aspects of its postcranium (MacLatchy, 2004; Nakatsukasa, 2008; Ward, 2015) and could possibly be an orthograde, the assumption that it might have been a suspensory ape mainly derives from the finding of a fragment of scapula preserving the glenoid cavity and scapular neck (MacLatchy et al., 2000). The glenoid fossa is oval and overall shallow, it lacks a notch in the craniodorsal surface of the glenoid margin (perimeter) and a lip, and the presence of glenoid labrum and proximal origin of the scapular spine is inferred (MacLatchy, 2004), thus resembling extant apes and the spider monkey. However, as argued above, functional and locomotor inferences derived from glenoid cavity morphology alone must be made with great care (or not made at all), especially if such claims cannot be supported by other elements of the forelimb that, as seen, might be better suited for it. Therefore, the claim that *M. bishopi* was a suspensory ape cannot be sustained on the basis of the morphology of its glenoid cavity alone.

There is, unfortunately, a gap in the African Miocene ape postcranial record from the Middle Miocene (ca. 16–11.6 Mya) until the advent of extant great apes (gorillas and chimpanzees) ca. 8–6 Mya. There is, however, at least one ape that shows adaptations toward enhanced forelimb‐dominated behaviors (nonsuspensory but heightened respect to earlier forms; Ishida, Kunimatsu, Takano, Nakano, & Nakatsukasa, 2004; Ishida et al., 1999; Nakatsukasa & Kunimatsu, 2009; Nakatsukasa, Yamanaka, Kunimatsu, Shimizu, & Ishida, 1998; Senut et al., 2004) and for which we have partial shoulder girdle remains. *Nacholapithecus* (ca. 15 Mya, Kenya) is well‐known from a multitude of remains and, especially, from the adult partial skeleton KNM‐BG 35250 (Ishida et al., 2004). This individual shows an unusual combination of features unlike any other (extant or extinct) ape (Ishida et al., 2004; Nakatsukasa & Kunimatsu, 2009; Ward, 2015) in being an overall pronograde but with adaptations resembling extant apes, probably related to the de‐emphasis of lumbar flexion–extension (dorsal stability; Nakatsukasa & Kunimatsu, 2009). The forelimb of *Nacholapithecus* shows mosaic characters (mixture of primitive and derived characters) but, unfortunately, there are no complete proximal humeri known for this taxon, and only scapular and clavicular features are known for the shoulder girdle (Ishida et al., 2004; Senut et al., 2004). The glenoid fossa is pear‐shaped and large and the acromion is projected beyond the glenoid, as seen in arboreal primates, and the clavicle is long and slender. These features, combined with a narrow trunk, suggest that the scapulae were laterally positioned, with the long clavicles either positioned in a cranial angle (as seen in orangutans) or with a proportionally large upper thorax with large muscles (Senut et al., 2004). The long clavicle with ligament insertions also suggests that protraction of the humerus in overhead postures would have been emphasized over abduction (Senut, 2003; Senut et al., 2004), with clambering and nonstereotypical arboreal behaviors (bridging, reaching, hoisting, transferring) being put forward as locomotor modes for this ape.

During the Middle Miocene the first apes appear in Eurasia, their dispersal bringing about a diversification in the range of locomotor repertoires not seen to date. There are evidences of, for example, the persistence of generalized locomotor behaviors, as seen in the Middle‐Late Miocene ape *Sivapithecus* (ca. 12–7 Mya; Madar, Rose, Kelley, MacLatchy, & Pilbeam, 2002; Pilbeam, Rose, Barry, & Shah, 1990); the appearance of the first undisputed orthograde (*Pierolapithecus catalaunicus*, ca. 15 Mya, Spain; Moyà‐Solà, Köhler, Alba, Casanovas‐Vilar, & Galindo, 2004, 2005); and the advent of the first unequivocal evidence of orthogrady paired with suspensory behaviors in the Late Miocene ape *Hispanopithecus laietanus* (ca. 9.5 Mya, Spain; Almécija, Alba, Moyà‐Solà, & Köhler, 2007; Alba, Almécija, Casanovas‐Vilar, Méndez, & Moyà‐Solà, 2012; Moyà‐Solà & Köhler, 1996; Pina, Alba, Almécija, Fortuny, & Moyà‐Solà, 2012). It is unfortunate that there are no glenohumeral remains preserved for any of these apes (but if there were, their glenohumeral remains should be expected to support the locomotor repertoires inferred for them from other postcranial remains).

**Figure 5:**
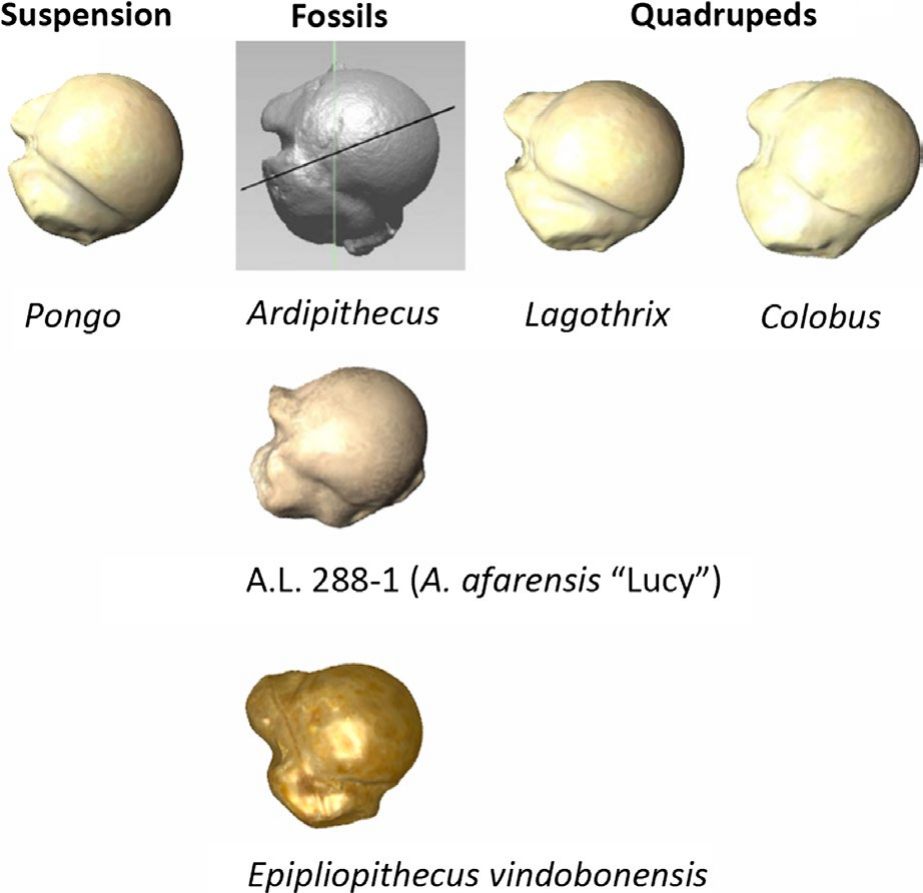
Comparison between fossil and extant primates proximal humeral morphologies, including the Middle Miocene pliopithecus *Epipliopithecus vindobonensis*, and the hominins A.L. 288‐1r (*A. afarensis*) and ARA‐VP‐7/2‐A (*Ardipithecus ramidus*). Note the striking similarity between *Ardipithecus* and *Pongo* (Image of *Ardipithecus* modified after Lovejoy et al., 2009a)

The study of the glenohumeral remains from nonhominoid extinct Eurasian primates is of help in answering central questions regarding the morphological pathways to the acquisition of suspensory behaviors and orthogrady (Arias‐Martorell et al., 2015b). Pliopithecoids, a group of extinct primates that inhabited Eurasia from ca. 18 to 7 Mya, were initially considered to be related to hylobatids due to superficial resemblances such as relatively small body mass or slender forelimb long bones (e.g., Hürzeler, 1954; Zapfe, 1958). However, pliopithecoids retain primitive features indicating a much earlier divergence (Begun, 2002; Harrison, 2005, 2010). *Epipliopithecus vindobonensis* (Early Middle Miocene, Slovakia) is one of the best‐known pliopithecoids, with abundant postcranial remains from various individuals (Zapfe, 1958). The locomotor repertoire of *E. vindobonensis* has been diversely interpreted, from an arboreal generalist and terrestrial quadruped to an agile above‐branch walker and runner displaying significant climbing, as well as hind limb and forelimb suspension (Fleagle, 1983; Harrison, 2013; Rein et al., 2011; Rose, 1983, 1989, 1994; Zapfe, 1958). Recent studies (Rein et al., 2011) reemphasized the importance of quadrupedalism in this taxon by quantitatively reexamining various characters of its forelimb (length of the olecranon process relative to the size of the ulna). However, the relative high degree of humeral torsion of *E. vindobonensis* would predict a low frequency of quadrupedalism—a combination most similar to that displayed by New World suspensory monkeys—whereas phalangeal curvature would support a significant amount of climbing behaviors as well (Rein et al., 2011). Shape analyses undertaken on the proximal humerus of *E. vindobonensis* indicate that it has its closest morphological affinities with the woolly monkey (Arias‐Martorell et al., 2015b). *E. vindobonensis* exhibits, like the woolly monkey, a fairly globular articular surface, rounder on the superior aspect of the articular surface compared to the other arboreal quadrupeds (capuchins and colobus monkeys), but clearly differing from the protruding and extremely globular articular morphology of extant apes and the spider monkey (Figure 5). The tubercles of *E. vindobonensis* also closely resemble generalized arboreal quadrupeds (Arias‐Martorell et al., 2015b), particularly a round *subscapularis* insertion, which stresses the role of this muscle as a powerful internal rotator and stabilizer of the joint during the quadrupedal gait (Larson, 1988, 2007a), as well as relatively large tubercles with respect to the articular surface, as seen in more quadrupedal taxa.

Although its main positional behavior would have consisted of generalized arboreal quadrupedalism, *E. vindobonensis* shows some forelimb suspensory adaptations, quite like the generalized (but still suspension‐capable) woolly monkey. From a wide‐ranging evolutionary perspective, the presence of these two features highlights the decoupling between the acquisition of suspensory adaptations (at least, at the glenohumeral joint) and that of an overall orthograde body plan, as the latter is lacking in both taxa. Both *E. vindobonensis* and the woolly monkey display proximal humeral morphologies enabling greater circumduction ranges in overhead limb positions than those of generalized arboreal quadrupeds, and as such, a fair amount of forelimb suspensory behaviors, without a concomitant shift toward an overall orthograde morphotype in torso and lumbar spine morphology (Fleagle, 1983; Harrison, 2013; Rein et al., 2011; Rose, 1983; Rose 1988; Rose, 1994; Rose et al., 1992). This suggests that the evolution of some suspensory adaptations, superimposed to an otherwise pronograde body plan suitable for generalized arboreal quadrupedalism (as in *E. vindobonensis*), might have been more common among extinct catarrhines than is generally assumed.

The presence of suspension has been traditionally linked to a series of derived morphological traits known as orthogrady, including a broad and shallow thorax, spinal invagination, long clavicles, dorsally placed scapulae with laterally oriented glenoid fossae, highly mobile shoulder joints, ulnar deviation of the hand, lack of ulnocarpal joint, a short lumbar column with dorsally placed transverse processes, visceral fixation, and loss of an external tail (e.g., Andrews & Groves, 1976; Gebo, 1996, 2010; Keith, 1903, 1923; Ward, 2015; Williams, 2012). However, with the expanding record of Miocene and Plio‐Pleistocene hominoid and pliopithecoid fossils, questions have been raised as to (a) the overall homology of those traits, exclusive of hominoids, thus constituting their morphological ancestral condition (Crompton, Vereecke, & Thorpe, 2008; Gebo et al., 1997; MacLatchy, 2004; MacLatchy et al., 2000; Williams, 2012), and (b) the assumption of presence of suspensory behaviors when orthograde features are recognized (mostly in the fossil record) and vice versa (Almécija, Alba, & Moyà‐Solà, 2009; Moyà‐Solà et al., 2004, 2005).

Regarding homology, the early hominoid *Morotopithecus* reviewed above poses two interesting scenarios for the evolution of orthogrady, depending on its phylogenetic position. If *Morotopithecus* is more closely related to crown hominoids (all the apes that would share a common ancestor), to the exclusion of the other Early to Middle Miocene African apes (*Afropithecus*,* Proconsul*,* Nacholapithecus*), the “orthograde” body plan would have been acquired as far as 20 million years ago, and would certainly be a crown hominoid synapomorphy (a derived characteristic shared by all subsequent members of the group; Gebo et al., 1997; MacLatchy et al., 2000; MacLatchy, 2004). However, if this is the case, the primitive condition of a pronograde body plan exhibited by the later ape *Sivapithecus*, which is regarded as a direct ancestor of orangutans based on cranial evidence, would have to be explained as an independent acquisition, having “re‐evolved” orthogrady later (as orangutans are orthogrades). The alternative explanation would be that *Morotopithecus* is not more closely related to crown hominoids than to any other particular taxon (i.e., it is a sister taxon to crown hominoids, not its common ancestor), and it would become an example of independent acquisition of orthogrady in general, and an evidence of homoplasy (in relation to orthogrady) within the ape lineage (Harrison, 2002, 2005). To this regard, morphological differences between the lesser and great apes have been interpreted as suggesting that suspension evolved in parallel in gibbons and siamangs and great apes (Larson, 1998). This seems to be further supported by the more primitive morphology (nonsuspensory) displayed by early members of the crown group (e.g., *Pierolapithecus*) or *Sivapithecus* (Alba, 2012; Madar et al., 2002; Moyà‐Solà et al., 2004; Pilbeam et al., 1990). This second scenario is even further supported by the glenohumeral evidence of the spider monkey and the apes, which show convergent suspensory adaptations (Arias‐Martorell et al., 2015a; Larson, 1998).

Regarding the correlation between suspensory behavior and orthogrady, the fact that all extant apes practice to some extent both vertical climbing and below‐branch suspension (Hunt, 1991a, 2004) has led to conflicting hypotheses on the adaptive role of suspension in the origin of orthograde features, with some authors stressing it (Gebo, 1996) and others favoring vertical and/or cautious climbing instead (Cartmill & Milton, 1977; Sarmiento, 1995). As discussed above, suspension has been further inferred for some extinct catarrhines retaining a pronograde body plan, most notably *E. vindobonensis*. On the opposite side of the spectrum is the case of *Pierolapithecus*, which displays a torso morphology that reflects an orthograde body plan, but lacks some key suspensory adaptations in the forelimbs, further suggesting a decoupling of the two features (Almécija et al., 2009; Moyà‐Solà et al., 2004, 2005).

To date, then, the earliest evidence for suspensory adaptations coupled with an orthograde body plan in the hominoid fossil record corresponds to *Hispanopithecus laietanus* (Alba, 2012; Alba, Almécija, & Moyà‐Solà, 2010; Alba et al., 2012; Almécija et al., 2007; Moyà‐Solà & Köhler, 1996; Susanna, Alba, Almécija, & Moyà‐Solà, 2014). As seen, *Morotopithecus* could challenge the latter statement, seeing that this ape seemingly exhibits orthograde features in the lumbar vertebrae coupled with suspensory adaptations (MacLatchy et al., 2000; Nakatsukasa, 2008; Sanders & Bodenbender, 1994). However, its postcranium would more likely suggest an instance of independent evolution of orthogrady (Alba, 2012; Harrison, 2010; Ward, 2015), based on the fact that (a) the evidence for suspension is drawn from the glenoid cavity alone and the issues that entails (it is worth keeping in mind that the morphology hailed as suspension‐diagnostic for primates is also found in cursorial mammals such as the horse; Roberts, 1974), and that (b) this ape exhibits an afropithecus‐like facial morphology (lacking hominid facial synapomorphies; Harrison, 2010).

Thus, based on current undisputed evidence available, orthogrady and suspension would have independently arisen several times throughout ape evolution. Among large‐bodied apes, the acquisition of an orthograde body plan seems to have taken place first (probably originally related to vertical climbing), with a later acquisition of suspension (which would have even appeared independently in some great ape lineages; Alba, 2012; Almécija et al., 2007, 2009; Cartmill, 1985; Crompton et al., 2008; Fleagle, 1976; Nakatsukasa, Kunimatsu, Nakano, Takano, & Ishida, 2003; Moyà‐Solà et al., 2004, 2005; Sarmiento, 1998). In contrast, small‐bodied primates (including extinct catarrhines such as *E. vindobonensis*) seem to have followed the reverse path, with suspensory adaptations being acquired on an otherwise pronograde body plan, which poses an interesting scenario for the evolution of gibbons and siamangs. The small size of these apes has been hypothesized to have been brought about through a process of dwarfism (e.g., Pilbeam & Young, 2004), but there are virtually no remains for lesser apes ancestors other than *Yuanmoupithecus* (ca. 7–8 Mya, China; Pan, 2006; Harrison, Ji, & Zheng, 2008), which is still largely unknown. This, then, does not exclude the possibility that they evolved from small‐bodied, pronograde stem hominoids, similar perhaps to *E. vindobonensis*, with their orthograde and suspensory adaptations having evolved in parallel with those of great apes. A new fossil (*Pliobates cataloniae*, 11.6 Mya, Spain; Alba et al., 2015) from the rich site of Abocador de Can Mata (Sabadell, Spain) has even brought its discoverers to put forward the hypothesis that the last common ancestor of crown hominoids might have been more gibbon‐like (or small‐bodied, generally quadrupedal but displaying use of forelimb suspension to some degree) than previously assumed.

The Miocene, thus, remains a fascinating period where ape locomotion diversified beyond any subsequent or past scope, and where the basis of the morphological changes that would finally lead to the rise of hominins and, ultimately, humans, were set.

## 6 EVOLUTION OF HOMINOID GLENOHUMERAL MORPHOLOGY: HOMININS

The fossil gap in Africa unfortunately covers part of the later Miocene period (ca. 10–8 Mya), when gorillas diverged from the hominin lineage to the chimpanzee–human split (ca. 6–5 Mya). Evidence available to characterize the last common ancestor (LCA) between humans and chimpanzees is that of the Late Miocene/Pleistocene putative early members of the hominin lineage: *Ardipithecus*,* Shaelanthropus,* and *Orrorin*. The debate has been mainly centered in the identification of bipedalism in each of these taxa (e.g., Almécija et al., 2013; Lovejoy, Suwa, Spurlock, Asfaw, & White, 2009c; White et al., 2009; Zollikofer et al., 2005), from which the bipedalism of latter hominins could have evolved, but in at least one case, there are known glenohumeral remains (although, unfortunately, not available for study to the wider scientific community; Lovejoy et al., 2009c; White et al., 2009).

The postcranium of the putative hominin *Ardipithecus*—as described by Tim White's team in a series of studies (White et al., 2009)—from the early Pliocene of Ethiopia (ca. 5.5–4.4 Mya), exhibits a modern‐looking pelvic morphology suggestive of habitual facultative bipedalism. The foot exhibits an amalgam of primitive features with specialized traits for habitual bipedality (Lovejoy et al., 2009c; White et al., 2009), the elbow shows full range of flexion–extension but lacks suspensory characters, and the hand exhibits adaptations consistent with above‐branch palmigrade behaviors (Lovejoy, Simpson, White, Asfaw, & Suwa, 2009a; Lovejoy, Suwa, Simpson, Matternes, & White, 2009b; White et al., 2009). Overall, the locomotor behavior of *Ardipithecus* is described as bipedalism with a large arboreal component, mainly above‐branch palmigrade quadrupedalism, clambering and bridging, resembling other Miocene taxa (especially *Nacholapithecus*), without the presence of suspension (Lovejoy et al., 2009a,b; White, Lovejoy, Asfaw, Carlson, & Suwa, 2015; White et al., 2009). There is at least one well‐preserved proximal humerus (ARA‐VP‐7/2‐A; Lovejoy et al., 2009a; White, Suwa, & Asfaw, 1994) which shows, according to the scholars that studied the remains, typical hominid characters, including “an elliptical head and shallow bicipital groove” (Lovejoy et al., 2009a; : 70e6), plus a minimum amount of torsion. Seeing how the proximal humeri of extant and extinct hominoids differ (however subtly) among taxa and how that reflects on locomotor behavior to a great extent, a detailed analysis of the proximal humerus of *Ardipithecus* would most likely render a wealth of information. For what can be qualitatively observed from the images provided in the publications of the remains (Figure 5), the morphology of the proximal humerus of *A. ramidus* appears well‐rounded and protruding in its articular surface, with tubercles appearing (to the naked eye) to be something in between those of the orangutan and the woolly monkey—as seen above in this review, both suspensory taxa, albeit being in opposite extremes of the spectrum of suspension‐using taxa. At the very least, the images provided for this proximal humerus would suggest a great range of circumduction at the shoulder level and possibly well‐developed arm‐rising capabilities. This would, of course, have to be tested conducting the adequate analysis of the remains to either confirm it or reject it, but such analysis is not possible at the moment.

Australopiths are one of the best represented and studied early hominin clades ranging from roughly 4 to 2 Mya, from *Australopithecus anamensis* to *A. sediba*, and covering territories from East Africa through South Africa. Among the australopiths, *A. afarensis* (ca. 4–3 Mya, Eastern Africa) is particularly well represented, followed by *A. africanus* (ca. 3–2 Mya, South Africa) and the more recent *A. sediba* (ca. 2 Mya, South Africa; Berger et al., 2010; Ward, 2013). Overall, the postcranial evidence clearly points to the hypothesis that committed bipedalism was their main locomotor behavior when on the ground, with fully orthograde bodies, but retaining the presence of arboreal traits in the forelimbs—including high intermembral and brachial indices, long and curved manual phalanges and a cranially oriented glenoid fossa (high glenoid‐bar index). The adaptive significance of such arboreal traits is highly debated, however, with some authors arguing in favor of australopiths being partly arboreal (exhibiting vertical climbing behaviors or even suspension; Jungers, 1982, 1991; Jungers & Stern, 1983; Larson, 1988, 2007a, 2012, 2013; Rein, Harrison, Carlson, & Harvati, 2017; Rose, 1984, 1991; Senut, 1980; Stern, 2000; Stern & Susman, 1981, 1983; Susman & Stern, 1991; Susman, Stern, & Jungers, 1984), exhibiting a compromise behavior of bipedal progression and some arboreality stemming from the retained arboreal characters (Cartmill & Schmitt, 1996; MacLatchy, 1996; Stern, 1999; Susman et al., 1984), or interpreting the arboreality‐related morphology of the forelimbs as primitive retentions without adaptive significance (Tardieu & Preuschoft, 1996; Ward, 2002, 2013).

Arias‐Martorell et al. (2015c), in an analysis of the proximal humeral morphology of one of the best‐preserved *A. afarensis* individuals, A.L. 288‐1 (“Lucy”), showed that the left humerus (A.L. 288‐1r) of this australopith female shows mixed characteristics between the derived condition of humans and a more generalized arboreal pattern. The analysis included another two australopith representatives, Sts 7 (*A. africanus*) and Omo 119‐73‐2718 (*Australopithecus* sp.), which also showed mixed arboreal traits, combining some orangutan‐like features with more generalized characteristics resembling the woolly monkey (especially Omo 119‐73‐2718). The patterns found in the proximal humerus would have indeed enabled these specimens to use a relatively significant amount of below‐branch positional behaviors (e.g., Larson, 2007a, 2013; Rose, 1991; Senut, 1980; Stern, 2000; Susman et al., 1984). None of the three australopiths shared the morphological condition of the African great hominoids (*Gorilla* and *Pan*), thus building on the contention that the LCA between hominins and panins could have exhibited a more generalized arboreal locomotor repertoire, instead of knuckle‐walking. Both A.L. 288‐1 and Sts 7 preserve the glenoid cavity of the scapula, hence morphological analyses of both were attempted, rendering, unfortunately but unsurprisingly, equivocal results (Arias‐Martorell et al., 2015c). In general, the glenoid cavities of both specimens showed more affinities to the great hominoids. The generally cranial orientation of the glenoid facets of these hominins (measured repeatedly in studies for Sts 7 and estimated for A.L. 288‐1; Campbell, 1966; Larson, 2007a; Oxnard, 1968; Robinson, 1972; Stern & Susman, 1983; Vrba, 1979), pattern also found in the juvenile *A. afarensis* scapula DIK‐1‐1 (Alemseged et al., 2006; Green & Alemseged, 2012) as well as in *A. sediba* (specimen MH2; Churchill et al., 2013), coupled with several other shoulder girdle characteristics (laterally placed supraglenoid tubercle, an ape‐like angle between the scapular spine and the axillary border, and a clavicle that lacks the characteristic human curvature), indicate that these hominins maintained a high shoulder position in a funnel‐shaped thorax. Such characteristic further suggests the capacity of sustaining abducted positions of the arm without the need of rotating the scapula upwards after the first 90 degrees of arm abduction, like the suspensory hominoids, putting these early hominins at an advantage position for niche exploitation. Thus, their locomotor behaviors would have consisted of full adaptation to bipedal terrestriality while on the ground, and to suspension/climbing while on the trees (Sylvester, 2006), with possible variations of the amount of suspension displayed (based on both its glenoid and scapular shape as well as other forelimb elements—e.g., ulnar shape—it has been argued that *A*. *sediba* was more suspensory than *A. afarensis*, for example; Churchill et al., 2013; Rein et al., 2017). The recently published partial skeleton of *A. afarensis* KSD‐VP‐1/1 (3.6 Mya, Ethiopia; Haile‐Selassie & Su, 2016) preserves most of the right scapula and is described by the authors as having a cranially oriented fossa as well (Melillo, 2016; Ryan & Sukhdeo, 2016). Melillo's (2016) analysis on shoulder girdle morphology (including qualitative observations, traditional metrics and geometric morphometrics) suggests that, when compared to all the other hominoids, *A. afarensis* shows more affinities to orangutans than any other group, and that its morphology is departed from that of the gorilla–chimpanzee cluster, making it highly unlikely for the LCA to have exhibited an African ape‐like shoulder girdle, agreeing with what has become the majority view (but see Young, Capellini, Roach, & Alemseged, 2015 for a different view on scapular shape). However, it is subsequently proposed that given her reconstructed morphology of the chimpanzee–human LCA (from scapular and clavicular traits only of extant hominoids) and the intermediate morphological condition of certain aspects between modern humans and orangutans of the KSD‐VP‐1/1 shoulder girdle, the forelimb of *A. afarensis* would seem to have been functionally selected for manipulative functions over locomotor ones. While this may possibly be partly true, it is worth keeping in mind that in the study conducted by Arias‐Martorell et al. (2015c), australopiths did not only exhibit morphological affinities with humans and the arboreal hominoids, but displayed similarities with more generalized monkeys as well (particularly with the woolly monkey). This stresses the importance of contextualizing the debate about the morphological affinities of early hominins with the inclusion of more generalized primate taxa that better characterize the evolutionary background of the hominoid lineage. As most studies stand now, only extant ape comparison samples are used, but due to the mosaic nature of the postcranial configurations of hominins, such studies might render relatively limited morphofunctional inferences because of the modern‐hominoid contained comparative sample.

The advent of the *Homo* clade (ca. 2–3 Mya) did not seem to reciprocate a morphological shift toward the modern human‐like condition right away (Larson, 2007a). The earliest species (*H. habilis* and, possibly, *H. rudolfensis*, ca. 2–1.5 Mya) retained australopith‐like overall postcranial morphologies, even though tool‐making abilities and manipulative capabilities are already present in these species—although the shoulder girdle remains are scant for *H. habilis* and nonexistent for *H. rudolfensis* (Larson, 2009). Early *H. erectus* depicts a distinct morphology not quite australopith‐like, not quite human (Larson, 2007a, 2009; Larson et al., 2007). Although no proximal humeri are known for *H. erectus*, it would seem that their shoulder girdle (of at least KNM‐WT 15000, relatively well‐preserved despite being juvenile) combined comparatively short clavicles, low humeral torsion and a protracted (lateralized) scapula position with an overall modern‐looking scapula (no upward‐facing glenoid cavity) that would have been sitting on a barrel‐shaped thorax (unlike the funnel‐shaped thorax of hominoids). Moreover, Arias‐Martorell et al. (2015c) report that the glenoid cavity of KNM‐WT 15000 is not quite human‐like either; its morphology was unlike any of the studied taxa (which included all hominoids plus the New World monkeys), adding to the contention that early *H. erectus* had a distinct morphology of its own—either transitional, as per Larson (2007a, 2009) or mosaic, retaining primitive and modern‐looking traits to comprise a morphology not seen in any extant species (Arias‐Martorell et al., 2015c). The major shift toward the modern human morphotype seems to have occurred in *H. heildebergensis* (ca. 0.6–0.2 Mya, Eurasia and Africa) and *H. neanderthalensis* (ca. 0.4 Mya, Europe), as well as the possible ancestor of both, *H. antecessor* (1.2–0.8 Mya, Spain; Larson, 2007a, 2009).

The analysis of the *H. neanderthalensis* proximal humerus of Tabun 1 (Arias‐Martorell et al., 2015c) virtually showed the same morphotype than that of modern humans, exhibiting a lowered neutral position of the arm, as discussed above (Kapandji, 2007; Larson, 1995, 2007b). The Tabun 1 specimen shows reduced insertion sites for the rotator cuff muscles, maybe indicating an early decrease of the above‐discussed reliance on the active stabilizers of the glenohumeral joint and diminished importance of the arm abductors (Bello‐Hellegouarch et al., 2013; Larson, 1995, 2007a; Potau et al., 2009; Roberts, 1974).

Neanderthal glenoids seem to be most similar to those of orangutans (superoinferiorly elongated, again signifying a possible lack of functional signal), which could result from developmental differences between Neanderthals and humans (Di Vincenzo, Churchill, & Manzi, 2012; Macias & Churchill, 2015). Di Vincenzo et al. (2012) study found that the differences on glenoid morphology between species of the *Homo* genus are related to a differential degree of development between the centers of ossification of the glenoid (Scheuer & Black, 2004) due to an enlarged growth period in modern humans (Di Vincenzo et al., 2012), and therefore not related to locomotion or to any functional constraint at that.

To a number of researchers, morphology at the glenohumeral level (and the whole of the shoulder girdle) at this stage in human evolution seems to be geared toward throwing effectiveness (Roach, Venkadesan, Rainbow, & Lieberman, 2013), with a few characters being essential—namely, humeral torsion and laterally oriented glenoid cavities. However, humeral torsion and some of the claims that have been derived from it (Roach & Richmond, 2015; Roach et al., 2013) are highly controversial, mostly because there is no consensus on what truly constitutes humeral torsion and what exactly the term stands for (this issue is beyond the scope of this review; for some of the debate see Cowgill, 2007; Larson, 1996; Larson, 2007b; Larson, 2015b; Rhodes, 2006; Roach & Richmond, 2015; Roach et al., 2013 and referenced therein). It is interesting that humeral torsion seems to be driven by the lateral rotation of the lesser tubercle to allocate for the bigger articular surface of the humerus, instead of being a result of an effective torsion of the humeral shaft (Fleagle & Simons, 1982). According to Rose (1989), however, more extensive articular surfaces are a separate trait from tubercle migration, although both may be combined, and thus, expansion can be brought about by the migration of one or both tubercles, combination of which (both tubercles migrating in the same direction or being brought closer together) would play a role in the final amount of torsion displayed. It is remarkable that Larson (1998) found that gibbons display the lowest humeral torsion of all living hominoids, while showing well‐rounded, big and protruding humeral articular surfaces, which she suggested is a compromise between the need of repositioning the scapula onto the back of the ribcage and extreme positioning of the elbow when engaging in brachiation (especially during ricochetal brachiation), and thus low torsion might be associated with the display of suspensory locomotion.

Overall, hominin glenohumeral joint remains play a key part on answering important questions about the morphological pathways that led to the acquisition of bipedalism, truly a defining characteristic of becoming human. But not only that, it also helps to understand that not all that seems uniquely human might be, and that hominins were part of the morphological stream that includes the hominoids that preceded them, and with whom they share more than previously thought to be possible.

## 7 FUTURE DIRECTIONS

Areas of future research within glenohumeral morphology could be circumscribed to anatomical and evolutionary. On the anatomical side, there is still a lot we do not know about soft tissue function and the role of passive stabilization at the joint. The glenoid cavity of the scapula remains an understudied aspect of it, and a very interesting one at that from a morphological standpoint. It is a region where soft tissue could be playing a fundamental role on function, that is, the presence of the cartilaginous rim (labrum) surrounding the cavity completely changes its shape and depth (personal observation), and thus possibly, its functional properties. From an evolutionary perspective, there is still a lot to be said about the adaptive role of glenohumeral morphology, and to what extent it shaped the hominoids evolutionary history. Questions such as what was the locomotor behavior of the LCA between humans and chimpanzees, or what was the extent of the role suspension played in the early days of the hominins only the discovery of new fossils (especially of the critical period of 8–6 mya) and the proper analysis of old ones will tell.

## 8 CONCLUSIONS

The glenohumeral joint remains a highly interesting and informative aspect of the postcranial skeleton to attempt morphofunctional explorations of extant and extinct primate taxa. Detailed and in‐depth analyses of the features of this joint provide insights into primate locomotor abilities, as well as offering a framework for contextualizing the evolutionary history of the groups under study. The proximal humerus, in particular, is of great importance, as its external morphology seems to be driven by the functional demands of locomotion. On the contrary, the glenoid cavity of the scapula does not seem to offer great insights into function, and any such conclusions should be considered with utmost caution.

It is important that the glenohumeral joint can help shed light on the evolutionary history of primates and hominoids in particular, being of special interest when dealing with topics such as homoplasy, orthogrady, the evolutionary role of suspension, and the morphology and locomotor behaviors of LCA nodes. For instance, there seems to be a decoupling between orthogrady and suspension, as the Eurasian small‐bodied catarrhines and the South American woolly monkey show clear adaptations to suspensory locomotion at the shoulder girdle and specifically at the glenohumeral joint without the acquisition of an orthograde body plan. The reverse condition seems to occur in some of the large‐bodied crown hominoids (*Pierolapithecus*), where the lack of suspensory adaptations goes together with an orthograde body plan. This also evidences the possibility that orthogrady and suspension may have arisen independently several times during hominoid and primate evolution.

Early (putative) representatives of the human branch (*Ardipithecus*) might have shown a degree of below‐branch positional behaviors while still bipedal on the ground, as seen in later hominins (*Australopithecus*), although such contention will need to await an in‐depth analysis of the whole fossil record.

At last, it is important to stress that any attempt to elucidate early hominin morphology from postcranial remains and derive behavioral hypothesis from such attempts would greatly benefit from including a wider range of comparative taxa, from the semisuspensory New World Monkeys to extinct hominoids (fossil record permitting), as the morphological affinities of early hominins are not as clear‐cut extant ape‐like as might have been assumed in the past.

## CONFLICT OF INTEREST

None declared.

## AUTHOR CONTRIBUTION

JAM conceived and designed the study, and wrote the manuscript.
