# Supplementary figures and images for: The morphology and evolutionary history of the glenohumeral joint of hominoids: A review
Source: Ecol Evol. 2018 Dec 30;9(1):703–22. doi: 10.1002/ece3.4392 (PMC6342098; doi:10.1002/ece3.4392)

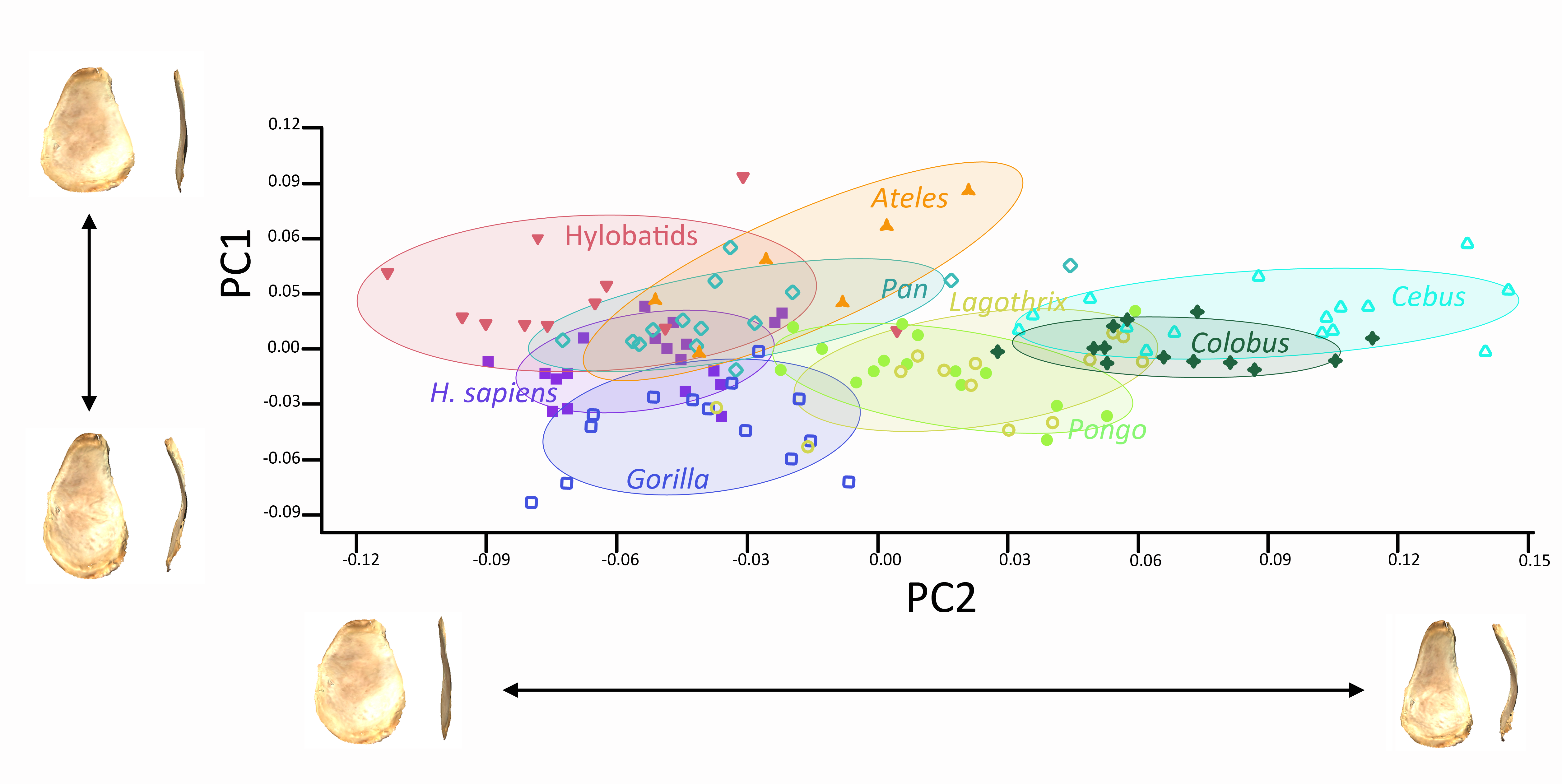

Supplement: Supplementary file 1 [file ECE3-9-703-s001.tif]
